# Social media quality in undergraduate medical education: A reconceptualisation and taxonomy

**DOI:** 10.1111/tct.13825

**Published:** 2024-11-06

**Authors:** Jonathan Guckian, Sarah Edwards, Eliot L. Rees, Bryan Burford

**Affiliations:** ^1^ Advanced Medical Education Fellow and Dermatology Registrar, Leeds Institute for Medical Education University of Leeds Leeds UK; ^2^ Emergency Department, Queen's Medical Centre University of Nottingham NHS Trust, NG7 2UH Nottingham UK; ^3^ Lecturer in Medical Education School of Medicine, Keele University Keele UK; ^4^ Senior Lecturer in Medical Education Newcastle University School of Medical Education, Newcastle University Newcastle upon Tyne UK

## Abstract

**Background:**

Social Media (SoMe) as a learning tool, though ubiquitous in society and popular within medical education, is often criticised as superficial. Its limitless output has been blamed for encouraging shorter attention spans and shirking in‐depth reflection. The evidence base is itself superficial and lacking rigour or meaning. We aimed to consider a theoretical basis for how ‘quality’ learning may happen on such platforms. Our findings then informed the construction of a taxonomy for SoMe learning.

**Methods:**

We conducted a qualitative interview study of United Kingdom (UK) medical students using a theory‐informed inductive study design. The research question was: ‘How do medical students conceptualise quality of learning on social media?’. We purposively sampled participants from responses to a short survey collecting demographic and SoMe usage data. Interview data were analysed using framework analysis and informed by Blooms taxonomy, connectivism and communities of practice (CoP) theories.

**Results:**

We received survey responses from 118 medical students across 25 UK medical schools. From these, 13 participants were recruited to individual semi‐structured interviews. We constructed three themes through framework analysis of interview data: cognitive hacking, professional identity reflection and safety, control and capital.

**Discussion:**

Quality SoMe learning may be conceptualised as a socially connected process, built upon constantly evolving networks but inexorably influenced by fluctuating hierarchy within learner‐centric CoP. Educators and institutions may support high‐quality learning for students through engagement which promotes community development, and safe, listening environments which foster professional identity formation.

## 1 BACKGROUND

Since its inception, Social Media (SoMe) for learning has been criticised as ‘superficial’.^1^ Its constant, limitless output has been blamed for ‘butterfly minds’, shirking in‐depth reflection.^2^ Yet, SoMe is pervasive across our way of life as society remains vulnerable to trends. SoMe popularity amongst medical undergraduates is beyond question: in 2011, before SoMe permeated professional life, 94% of United States (US) medical students were already reported to be users.^3^

SoMe has long been associated in the medical education literature as being successfully used for production of ‘bitesized’ learning materials, knowledge translation and debating research outcomes.^4^ Recent reviews have linked ‘good practice’ SoMe to community support, disseminating meeting pearls, and facilitating rapid, text‐based reflection.^5^, ^6^ These reviews are limited by an evidence base which is dominated by evaluative studies of single‐centre initiatives lacking rigour or meaning. There is lack of consensus as to whether SoMe is superficial, or if it can possibly lead to high‐quality learning in terms of outcomes or process. The purpose of this study was to examine how medical students conceptualise learning ‘quality’ on SoMe, to consider a theoretical basis for how ‘quality’ learning may happen on such platforms. Following this we aimed to construct a taxonomy for SoMe learning.

There is lack of consensus as to whether SoMe is superficial, or if it can possibly lead to high‐quality learning in terms of outcomes or process.

## 2 METHODS

### 2.1 Design

We conducted a qualitative study of UK medical students using a theory‐informed inductive data analysis design.^7^ We used Bloom's taxonomy, connectivism and CoP theories to inform data collection and analysis.^8^, ^9^, ^10^ First, we developed and distributed an online survey to UK medical students. Respondents to this survey were then purposively sampled for invitation to an individual interview. Since our completion, Twitter rebranded as ‘X’; however, we will continue to refer to it as Twitter to remain faithful to the context of the research.

#### 2.1.1 Theoretical framework

Within this study, we have used three learning theories to inform data collection and analysis. Firstly, Bloom's taxonomy helped conceptualise levels of quality.^8^ The cognitive domain of the revised taxonomy maps learning in a hierarchy, from ‘remembering’, through ‘understanding’, ‘applying’, ‘analysing’, ‘evaluating’ to ‘creating’. In the absence of robust objective interventional SoMe research, defining ‘quality’ in the context of medical education resources was challenging. We linked quality to excellence and as less ‘superficial’. Therefore, the application of Bloom's taxonomy allowed us to view data using a coherent theoretical lens whilst not obstructing learner perceptions on quality. Next, we used the learning theories we considered most relevant to SoMe education, including connectivism and CoP (Box 1).

Box 1 **Connectivism and CoP as applied to SoMe.**

**Connectivism** argues that learning lies in diversity of opinions, as human or non‐human ‘nodes’ are connected, designed in the context of online learning. SoMe features limitless capacity for such connections. The processes of choosing accounts to follow on Instagram, navigating Twitter polls and seeking alternate viewpoints from different learning environments are the embodiment of connectivism.^9^

**Communities of Practice** require groups with shared norms, activities and responsibilities in specific settings, with new learners moving into the community over time as ‘legitimate peripheral participants’.^10^ Closed SoMe platforms feature CoPs in action, with knowledge flowing freely amongst those with shared interests. This may be demonstrated amongst patient education populations on Facebook Groups, educators and students on Reddit and across international conferences on Twitter.

The application of Bloom's Taxonomy allowed us to view data using a coherent theoretical lens whilst not obstructing learner perceptions on quality.

### 2.2 Sampling and recruitment

UK medical students were first recruited via a survey, shared via Twitter, Facebook, Instagram and Reddit, asking questions on demographics, platform use, SoMe learning behaviours and open‐ended questions (Supplementary file 1). Questions regarding how participants use SoMe to remember, understand, apply, analyse, evaluate and create learning were asked. Our survey distribution strategy aimed to maximise reach and behaviour variation, though ultimately this was a convenience sample. As a sampling approach, this survey was not intended to capture the broad range of the UK medical student population's SoMe use. Instead, we wished to clarify the basics of behaviours and identify experienced medical student SoMe users to provide insights into quality across platforms during interview. Participants for interview were selected based on SoMe learning behaviours, use of specific platforms and basic perceptions applied to the taxonomy. Efforts were made ot ensure a range of year groups, universities, genders and ethnic backgrounds were represented.

### 2.3 Data collection

We conducted individual semi‐structured interviews, following a schedule based on a literature review of SoMe education and questionnaire responses (Supplementary file 2). Interview questions covered participants' experiences of learning using SoMe, perceptions of SoMe learning and how they defined quality in relation to SoMe learning resources. Interviews were all conducted by one researcher (NAME) online remotely using Zoom and audio recorded. The audio recordings were transcribed verbatim via Trint software and checked for accuracy by remaining researchers.^11^

### 2.4 Data analysis

We analysed interview data using framework analysis.^12^ Firstly, we familiarised ourselves with the dataset by reading through the transcripts. Next, a framework was constructed based on theory and issues identified during familiarisation. Transcripts were independently coded to the framework by three researchers to sort data for indexing. We developed a framework matrix, producing a summary for each theme for each participant based on original data. Summaries then facilitated ‘between‐participant’ analysis for each theme and ‘between‐theme’ analysis for each participant. Finally, we reviewed the full framework matrix and revisited the original data excerpts to explain relationships between categories.

#### 2.4.1 Reflexivity

Throughout the study, we were cognisant of the inevitability of our prior experiences and preconceptions influencing data collected and interpretations drawn. No authors had a direct working relationship with participants. [ANONYMISED] have all undertaken SoMe research, including both positive and detrimental influences. We endeavoured to remain reflexive through group discussions and considering whether data could be interpreted in different ways. While recognising limitations of member checking, we chose to undertake this in order to facilitate reflection on assumptions.^13^

#### 2.4.2 Ethics

Ethical approval was granted from Newcastle University Ethics Committee (18849/2019). This manuscript is reported in accordance with the Standards for Reporting Qualitative Research.^14^

## 3 RESULTS

**FIGURE 1:**
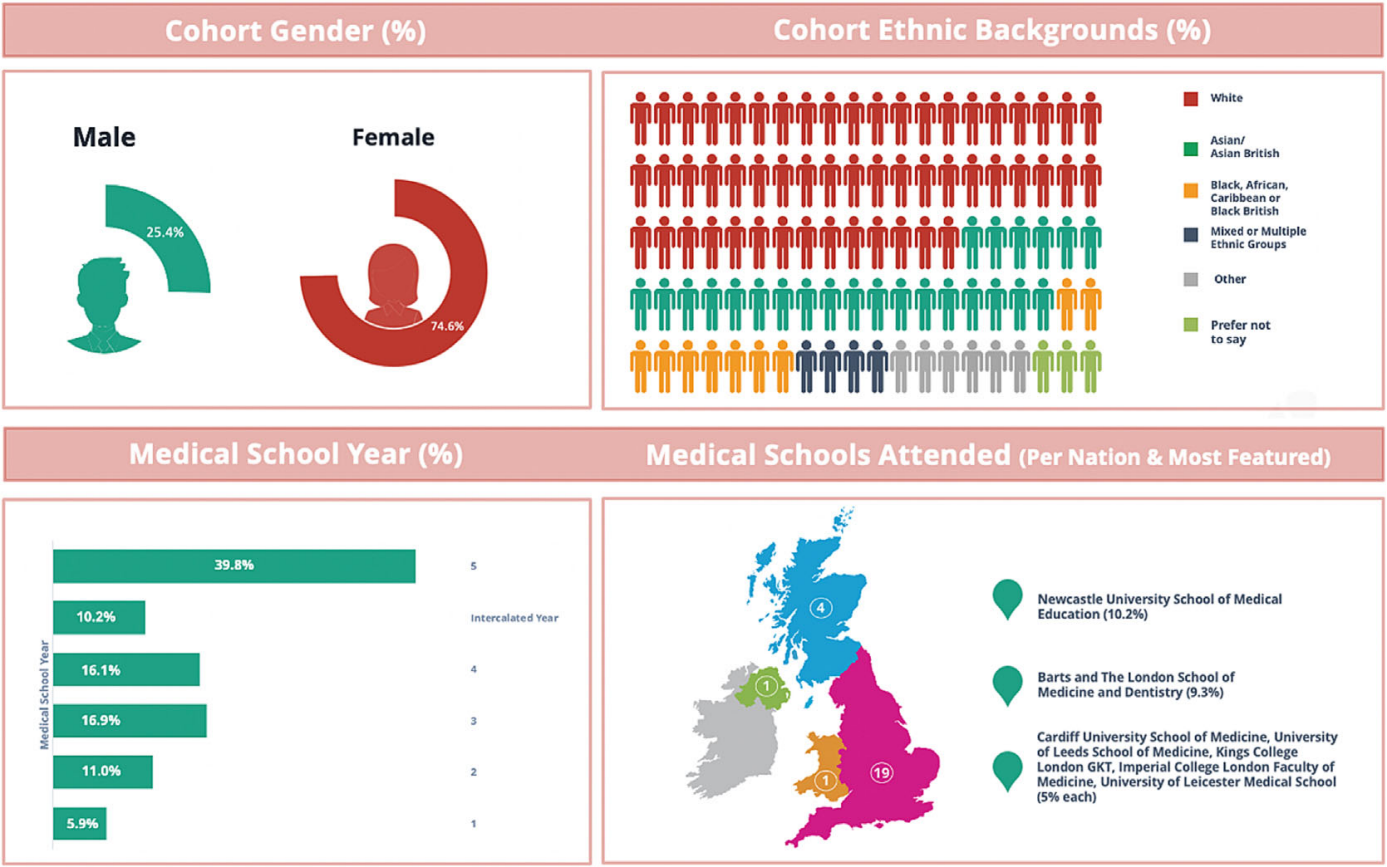
Questionnaire respondent cohort demographic breakdown.

One hundred and eighteen survey responses were recieved across 25 UK medical schools. Demographic details are outlined in Figure 1. A quarter (25.4%) of participants were male. Final year students represented a majority (39.8%) of participants followed by third year (16.9%), fourth year (16.1%), second year (11.0%), interalacting(10.2%) and first year students (5.9%).

This was a SoMe‐active population, with 13% of respondents using SoMe for 1–5 h per week, 26% 6–10 h, 23% 11–15 h, 14% 16–20 h, 12% 21–25 h and 12% over 25 h per week. For general use, Facebook and WhatsApp (both 99.2%) were most popular, followed by Messenger (96.6%), Instagram (90.7%), Twitter (89.8%) and Snapchat (72%). For learning activities, Twitter was most popular (71.2%), followed by Instagram (69.5%), Facebook (59.3%) and WhatsApp (40.7%).

Twenty respondents were purposively sampled for invitation to semi‐structured interviews. From this purposive sample, thirteen participated (Table 1).

**TABLE 1:** Demographic details of interview participants.

Interview no.	Gender	Ethnic background	Medical school year	Medical school region	Undergraduate or postgraduate entry	SoMe usage (hours/week)	Interview duration (min)
1	Female	White	3	West Midlands	Postgraduate	20–25	49:33
2	Male	White	Intercalating	Wales	Undergraduate	11–15	52:45
3	Female	White	5	London	Undergraduate	11–15	1:01:39
4	Female	White	3	Scotland	Postgraduate	>25	45:29
5	Female	Mixed or multiple ethnic groups	4	West Midlands	Postgraduate	6–10	1:01:36
6	Male	White	3	Scotland	Undergraduate	11–15	41:14
7	Female	Asian or Asian British	5	London	Undergraduate	11–15	51:59
8	Female	White	Intercalating	South‐East England	Postgraduate	>25	52:19
9	Female	White	3	Yorkshire & Humber	Undergraduate	20–25	35:57
10	Male	White	1	Scotland	Postgraduate	15–20	49:39
11	Female	White	5	Scotland	Postgraduate	11–15	1:00:07
12	Female	White	Intercalating	Northern Ireland	Undergraduate	11–15	59:37
13	Male	Black, African, Caribbean or Black British	5	South‐East England	Postgraduate	15–20	43:51

Through framework analysis, we constructed three themes: cognitive hacking, reflection on professional identity, and safety, control and capital.

### 3.1 Cognitive hacking

Medical students referred to SoMe as ‘chaotic’. An intimidating, vast, connectivist, atmosphere was described, where conflict was common. An overwhelming amount of superficial or irrelevant information contributed to cognitive load. Facing cognitive load, students developed strategies to provide themselves with the most useful learning and support wider community learning. We coined this process ‘cognitive hacking’, where ‘hacking’ was defined as ‘a process that makes another process easier’.

‘People come up with amazing ideas or again, hacks to memorise content'Participant 1, year 3, female.

Facing cognitive load, students developed strategies to provide themselves with the most useful learning and support wider community learning.

Cognitive hacking was a student‐led process, distinct from direct expert involvement. The need to ‘translate’ seemingly endless learning materials into a learner‐friendly format was due to the idea that educators or experts ‘lost sight of the basics’. This manifested in a learning environment filled with infographics, flashcards, brief video explanations and mnemonics. Cognitive hacking also concerned curation of higher‐quality learning environments. As students developed their SoMe accounts, they made conscious or subconscious decisions on who to follow, making value judgements on the worthiness of peers, experts or organisations.

‘Even while being on social media, I'm during my break time, enjoying my downtime, I'm relaxing. There's still the opportunity for me to learn something that I might not have otherwise had'Participant 13, year 5, male

### 3.2 Reflection on professional identity

SoMe exposed medical students to previously under‐recognised realities of life as a doctor. Social justice movements born on SoMe raised a sense of social responsibility, felt to be absent from traditional curricula, filling an important, high‐quality supplement to traditional curricula. Cultural and educational critique was fostered by SoMe medical communities as students felt empowered to change behaviours in positive ways.

‘And then it kind of opened up a bigger conversation, which did happen on social media […] is medical education racist, and like, are we inherently racist and not realising it?’Participant 12, intercalating, female

SoMe exposed medical students to previously under‐recognised realities of life as a doctor.

As part of their SoMe CoP, doctors were sought out for support in understanding realistic perspectives of working life, accessibly providing a ‘human conversation’ as ‘wisdom’ was sought from ‘elders’. However, it was felt that SoMe had ‘blurred the boundary’ between medical students and junior doctors, leading to a hierarchy flattening that enabled students to seek support.

‘It necessarily is more learning about how the medical world works, if that makes sense … someone telling me about shift patterns and things about and how rotas work, so all the things that you can't really gain from just like a book type thing, it's a human conversation'Participant 7, year 5, female

### 3.3 Safety, control and capital

Lack of safety was consistently linked with poor‐quality SoMe learning. Specifically, participants overwhelmingly viewed medical schools critically. This was so extreme that language used by numerous students described institutions as ‘abusive’ authorities. Students referred to their community as ‘battered’, ‘abandoned’ and ‘lost’. Students pronounced medical school as being ‘fight or flight’, leading to insecurities. One interviewee reported being told by a university leader to ‘keep my opinions to myself’.

‘Especially in the past year when we have been battered by medical school for the past years… But here's a handful of other people that are also finding it overwhelming and a lot to learn. And you can kind of you can wallow in your pain and extend your crisis together’Participant 10, year 1, male

Whilst students had been retreating to private SoMe learning communities for some time, disruption by the COVID‐19 pandemic accelerated this process. Learners adapted much quicker to chaos than faculty, described as conspicuous by their absence. A fear of ‘professionalism’ concerns from institutions drove students to practice lower‐quality learning behaviours, such as ‘lurking’, or even establishing anonymous or ‘burner’ accounts. Given learners associated quality learning with trustworthiness and accountability, such behaviours led to concern.

‘That comes with risks because you have to be careful about what you put out with the GMC hanging over everyone's shoulders’Participant 1, year 3, female

A fear of ‘professionalism’ concerns from institutions drove students to practice lower‐quality learning behaviours…

## 4 DISCUSSION

This study aimed to examine how medical students conceptualise learning ‘quality’ on SoMe. Three key themes were constructed: congnitive hacking, refelction of professional identity and safety, control and capital. Quality SoMe learning may be best conceptualised as a socially connected process, built upon constantly evolving networks but inexorably influenced by fluctuating hierarchy within learner‐centric CoP. Students take advantage of SoMe technology to cognitively process information into ‘better’ learning, to remodel professional identities and generate safe environments. Our results describe a range of rapidly evolving SoMe learning activities, and replicate recent findings concerning SoMe and positive professional identity formation in wider education.^15^ Moreover, the role of CoP in promoting safety as learning quality indicator has recently been highlighted in undergraduate medical education.^16^ Recalling the knowledge dimensions of Bloom's taxonomy more widely, it is possible to categorise SoMe learning activities (Table 2).

**TABLE 2:** A Bloom's taxonomy cognitive grid for social media, synthesised from this study's results.

	**Factual** *Basic information a learner must acquire to undertake higher quality learning*	**Conceptual** *Interrelationships between basic elements that can provide context & meaning*	**Procedural** *Awareness required to undertake specific skills*	**Metacognitive** *Awareness and knowledge of one's own cognition*
**Remembering**	List causes of clubbing through an Instagram flashcard	Recognise an account sharing mind‐maps on Pinterest	Recall cannula insertion technique from a TikTok video	Identify mnemonics to remember symptoms of thyroid disease
**Understanding**	Summarising case details in a Twitter thread	Interpret chest X ray findings from an Instagram post	Clarify ambiguous information through Facebook post comments	Predict which accounts will provide enjoyable learning content
**Applying**	Respond to an expert‐moderated question on Whatsapp	Ask appropriate clinical history questions in a live Twitter case	Deliver bad news during a Discord role‐play	Comment on a subreddit post regarding a topic of strength
**Analysing**	Organise clinical guidelines accessed through SoMe	Contrast accounts in a Twitter debate offering differing opinions	Follow accounts based on reliability	Deconstruct biases regarding education in skin of colour from Twitter and Instagram trends
**Evaluating**	Identifying role‐models on Instagram	Assess the relevance of critical comment responses	Draw conclusions on the veracity of possible Facebook misinformation	Reflect upon ‘professionalism’ of Twitter posts
**Creating**	Compile a collection of useful SoMe resources for community sharing	Assemble a group of peers to form a SoMe research collaborative	Write a Twitter quiz for junior medical students, featuring polls and data interpretation	Build a Whatsapp learning group of peers and experts to support each other's weaknesses

Quality SoMe learning may be best conceptualised as a socially connected process, built upon constantly evolving networks but inexorably influenced by fluctuating hierarchy within learner‐centric CoP.

Our findings demonstrate numerous examples of creation, the peak of the taxonomy, concerning learning activities and personalised learning environments. Whilst the literature frequently describes superficial‐appearing infographics or flashcards,^17^, ^18^ our findings suggest that these are actually digital footprints: evidence of higher quality, connectivist and community‐driven creation supported by SoMe environments. Connectivism, inherently built upon chaos, complexity and self‐organisation,^9^ explains how cognitive hacking leads to quality learning. Through cognitive hacking, creators navigate chaos and gather complex information from experts, translating this into brief lessons, to be processed for further creation. The existence of such learning involving constant flow of reflective information is not novel, with previous research linking rapid idea sharing on SoMe enhanced student performance.^19^ However, that 2014 study focused on negative consequences of SoMe, including significant distraction. Our results demonstrate that learners have embraced and crafted distraction as a tool for creation of individualised learning environments in the form of self‐curated feeds, adding to previous suggestions of distraction as a potentially positive learning tool.^20^ In applying connectivist principles, our results place connection at the forefront of quality, representing learning as a continuous, flexible, evolving process, driven by competition, community and individual need.^9^

Our results demonstrate that learners have embraced and crafted distraction as a tool for creation.

Tweetorials or ‘threads’ were common educational activities cited by students to encourage high‐quality critique. Their potential for supporting critical thinking has previously been highlighted in the literature.^21^ Whilst our study found that Tweetorials can facilitate understanding, more depth is required to promote the highest quality learning. Tweetorials were augmented by embedment into realistic clinical scenarios, generating debate or sparking reflection. These require community input to ensure quality, indicating evaluation is a social learning phenomenon occurring within CoP. Such CoP often influence learner perception of quality. For example, journal clubs have been highlighted in the literature as being a high‐quality educational activity.^22^, ^23^ However, these were derided by our respondents as inaccessible and ineffective, due to a culture of ego, competition and hierarchy.

Critique also included the nature of SoMe itself. Whilst studies^4^, ^24^ have highlighted the appeal of novelty and visual multimedia in driving SoMe acceptance, our findings stress that the reality is more complex. Medical students are not magpies to be drawn in by shiny new trends. Learners consistently critically appraise new information, curate specialised and complex personal learning feeds and are sceptical of information which does not fit existing worldviews, moving off SoMe to clarify authenticity. Students were intolerant of ego and self‐promotion, often perpetuated by ‘influencers’.^25^ Our study informed the synthesis of ‘quality indicators’ for SoMe learning, based on our themes (Table 3).

**TABLE 3:** Descriptive terms interview participants associated with ‘high‐quality’ SoMe learning.

Cognitive hacking	Professional identity reflection	Safety, power and capital
Concise/high yield	Diverse	Accessible
Community‐centric	Community‐centric	Community‐centric
Visually appealing	Narrative‐driven	Peer‐validated
Time‐valued	Popular	Expert supported
Creative	Realistic	Flattened hierarchy
Organised		Safe
Convenient		Non‐judgemental
		Appropriately challenging
		Anonymous

Our findings suggest that values formation was high‐quality, community‐centred and tied to group reflection. This has been previously examined as learners processed social backlash to a vascular surgery journal article, felt to be misogynistic in its assessment of trainee professionalism.^26^ Certain SoMe values appeared common within our participants, across platforms. These included community enablement, criticality, health advocacy and a constant desire for learning. Outside of professionalism, medical student SoMe values have been largely unexplored in the literature. Whilst this phenomenon is difficult to place within Bloom's cognitive domain, the affective domain associates quality with emotional development, including feelings, values and attitudes.^8^ Such individual and collective growth was prevalent across our results.

### 4.1 Implications for practice

These findings may offer guidance to educators on factors promoting higher quality SoMe learning. Brevity and collaborative outputs should be considered in augmenting any SoMe lesson plan or programme. Brevity has been linked to the convenience of learning ‘on the go. Our participants took this even further, by wielding SoMe learning as a ‘24/7’ tool to suit individual and collective agendas across time‐zones.^27^ Furthermore, collective learning has been associated with quality, with peer observation found to be a key feature of closed‐group learning.^28^ Our results have demonstrated that medical students not only observe each other, but also evaluate their own and each other's weaknesses to form small collaborative working groups for mutual development. The strength of community spirit goes beyond much of what has been described previously, though the looming threat of ‘toxic competition’ remained.

The strength of community spirit goes beyond much of what has been described previously, though the looming threat of ‘toxic competition’ remained.

Faculty development regarding SoMe resources may benefit from focusing on curating skills relating to our quality indicators (Table 3). It may prove challenging to push learners to specific levels within Bloom's taxonomy; therefore, educators might instead find success in cultivating programmes centred on the quality indicators, such as supporting community creativity, facilitating safe environments or providing expert validation. Lessons from this research have implications for wider health professional educators, given our undergraduate population is taught by a variety of professionals. Social media, for example, has been described as beneficial for professional identity development within nursing education,^29^ whilst SoMe CoP have been described amongst pharmacists and radiographers.^30^ Our findings add to such bodies of work. Concerning institutional responsibilities, efforts to repair what students alarmingly described as an ‘abusive’ relationship may manifest in the training of faculty and near‐peer role‐models in SoMe mentorship. Wellbeing support networks may provide benefit to those learners suffering from a competitive, ‘toxic’ SoMe environment.

### 4.2 Implications for research

Lessons may arise from this study to inform future research. Our community must advance from lower level, evaluative study of the latest SoMe trend, instead pursuing rigorous research answering meaningful questions with cross‐platform implications. Specifically, further research may consider Bourdieu's theory of habitus in exploring the consequences of popularity or social capital on quality in the SoMe learning environment.^31^ Research into both medical and wider healthcare professions educator and institutional perceptions of SoMe is warranted, as such perspectives are absent in this research, providing a one‐sided view.

Our community must advance from lower level, evaluative study of the latest SoMe trend, instead pursuing rigorous research…

### 4.3 Strengths and limitations

This study has several strengths. It was theory‐informed, applying Bloom's taxonomy as a theoretical framework through which to perceive data. Additional means of asserting rigour, including guideline‐based survey development, data triangulation, ‘insider’ inclusion within sampling, analysis transparency and member checking are strengths. The student‐focused nature of this study fills an important gap. Nevertheless, limitations are inevitable. Whilst efforts were made to ensure the study sampling was representative, some ethnic backgrounds, geographical locations and genders were underrepresented. Also underrepresented, by design, were perceptions of faculty or authority figures, which will introduce bias. Given the qualitative‐dominated approach to our research, transferability was not the aim of this study and we cannot claim that 118 students capture all relevant SoMe behaviours. Any representativeness that is apparent must only be relevant at the time of study, particularly given the rapid advancement of technology and societal trends. Therefore, newer platforms are absent, whilst significant recent changes to platforms are not considered.

## 5 CONCLUSIONS

Criticisms of superficial learning are themselves shallow concerning SoMe learning. Medical students navigate swathes of information to facilitate learning with remarkable ease, harnessing platforms to curate positive learning environments, model their future careers and support colleagues. They do so largely without formal institutional guidance and achieve high‐quality learning in domains neglected by formal curricula, including self‐reflection and critique of societal norms. SoMe is perfectly designed to enable creation and critical reflection, and this is most likely to occur when students feel safe and personal growth is fostered. It is the responsibility of educators and researchers to predict educational trends, rather than react too late.

SoMe is perfectly designed to enable creation and critical reflection, and this is most likely to occur when students feel safe…

## AUTHOR CONTRIBUTIONS

**Jonathan Guckian:** Conceptualization; investigation; writing—original draft; methodology; validation; visualization; writing—review and editing; software; formal analysis; project administration; data curation; resources. **Sarah Edwards:** Writing—review and editing; visualization; methodology; investigation; formal analysis. **Eliot Rees L:** Writing—review and editing; investigation; methodology; formal analysis; visualization. **Bryan Burford:** Supervision; conceptualization; investigation; methodology; formal analysis; writing—review and editing.

## CONFLICT OF INTEREST

The authors declare no conflict of interest. No patient consent or permissions to reproduce material from other sources are required.

## ETHICS STATEMENT

Ethical approval was granted from Newcastle University Ethics Committee (reference 18849/2019).

## Supporting information


**Supplementary File 1:** Study Questionnaire.

**Supplementary File 2:** Interview Schedule Guide.

## Data Availability

The datasets used and analysed during the current study are available from the corresponding author on reasonable request. No other papers are declared to be using the same dataset.
