# Supplementary figures and images for: Social media quality in undergraduate medical education: A reconceptualisation and taxonomy
Source: Clin Teach. 2024 Nov 6;22(1):e13825. doi: 10.1111/tct.13825 (PMC11663730; doi:10.1111/tct.13825)

**Supplementary File 1: Study Questionnaire**

# Appendix E: Study Questionnaire


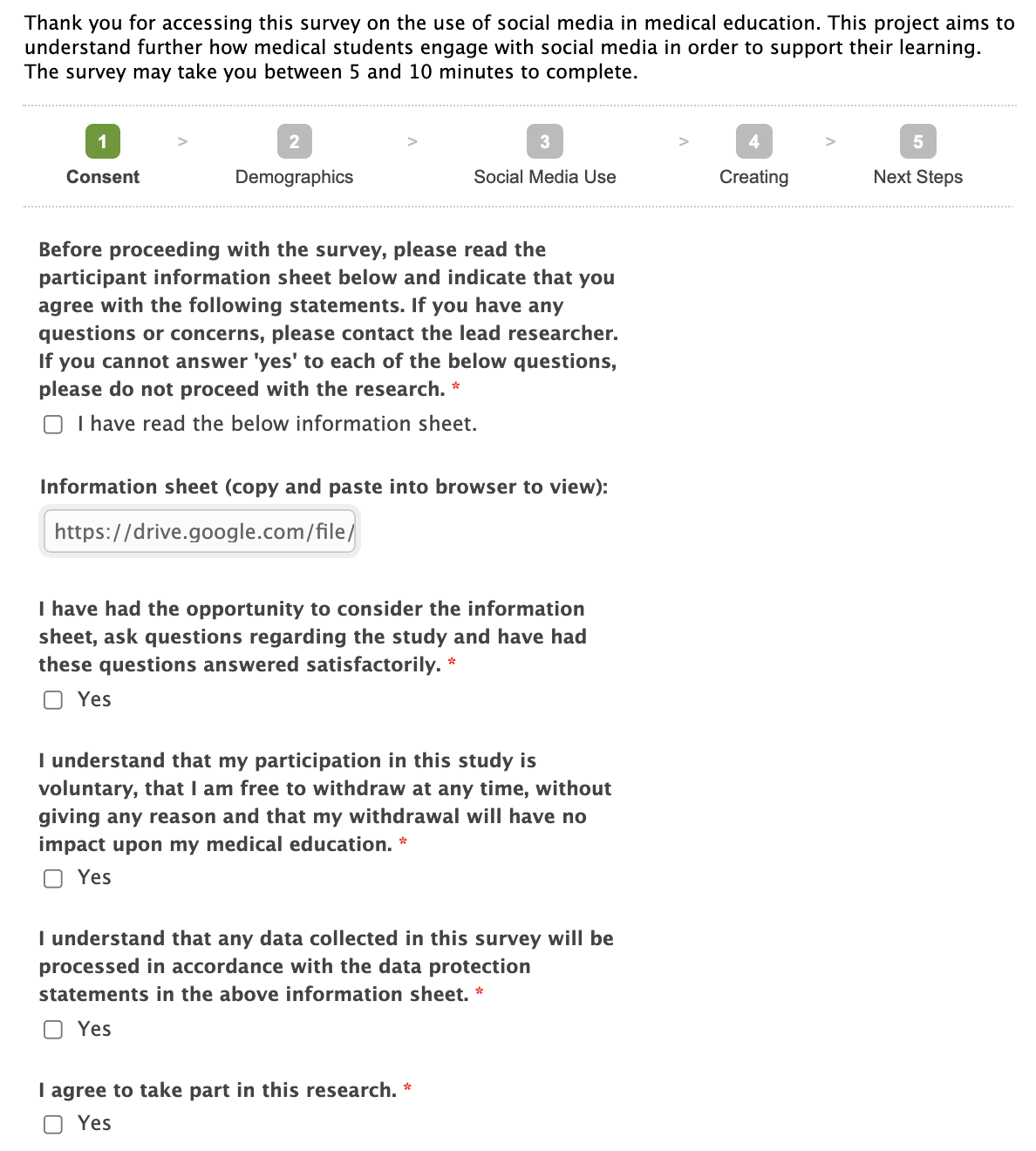


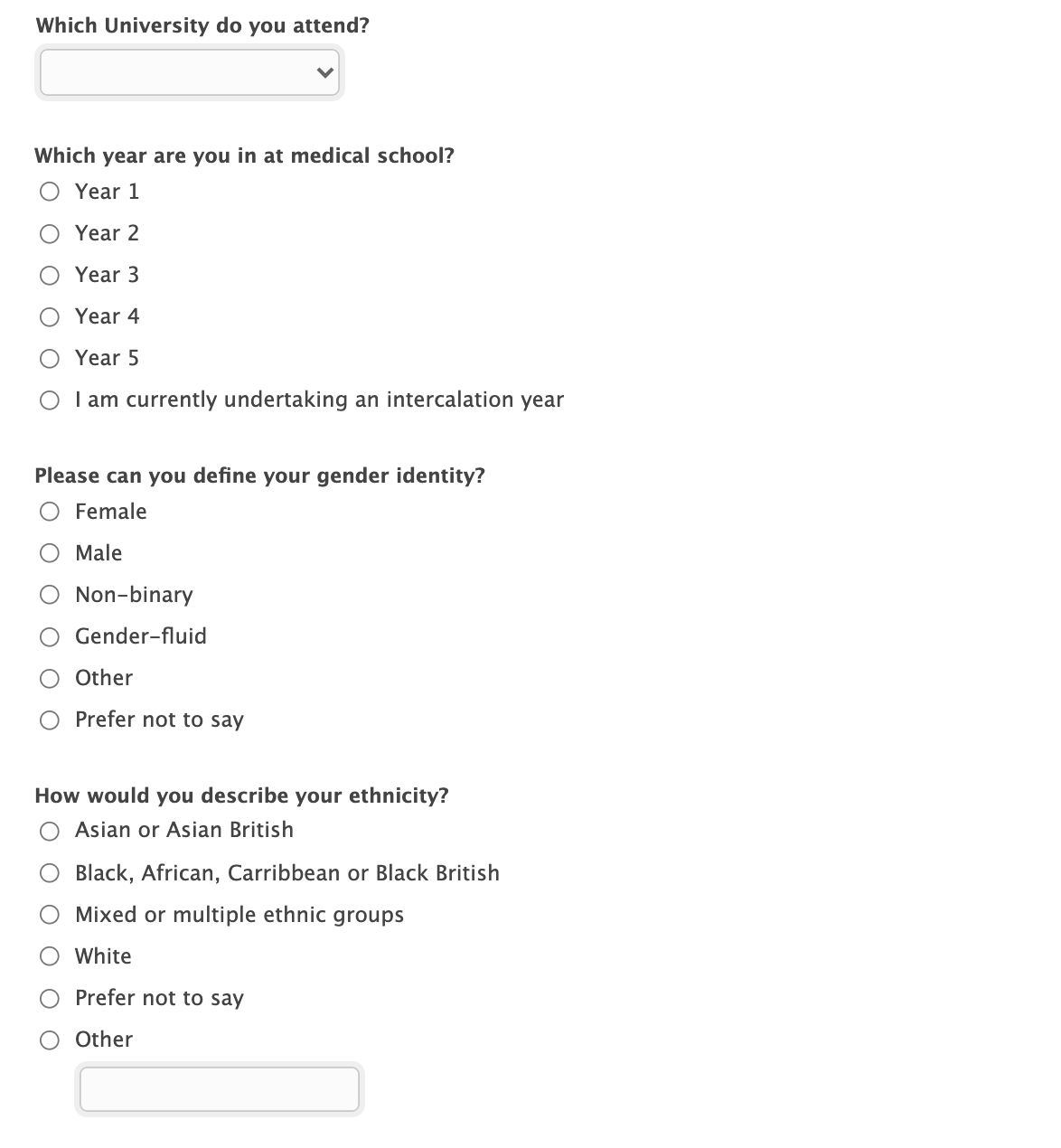


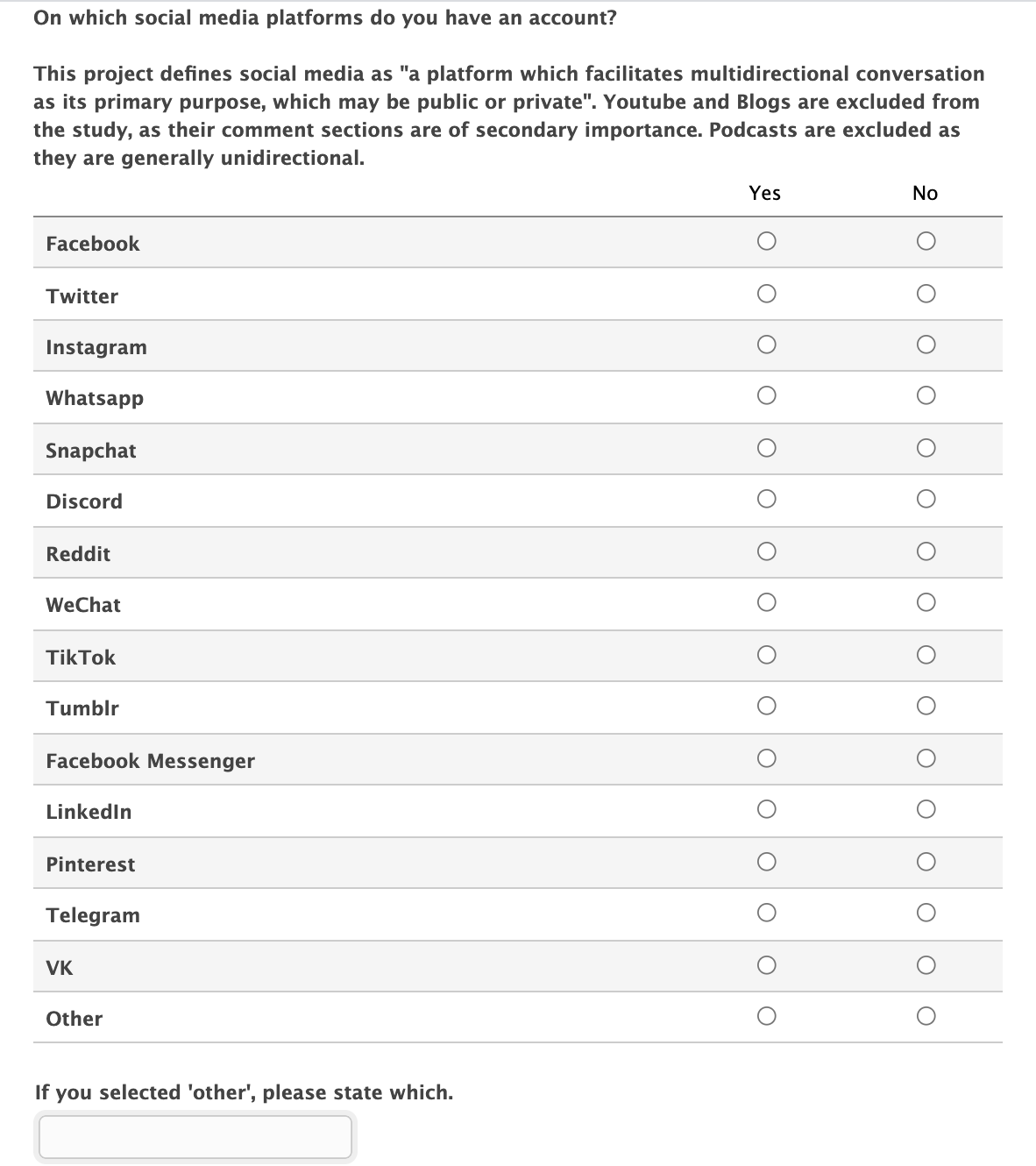


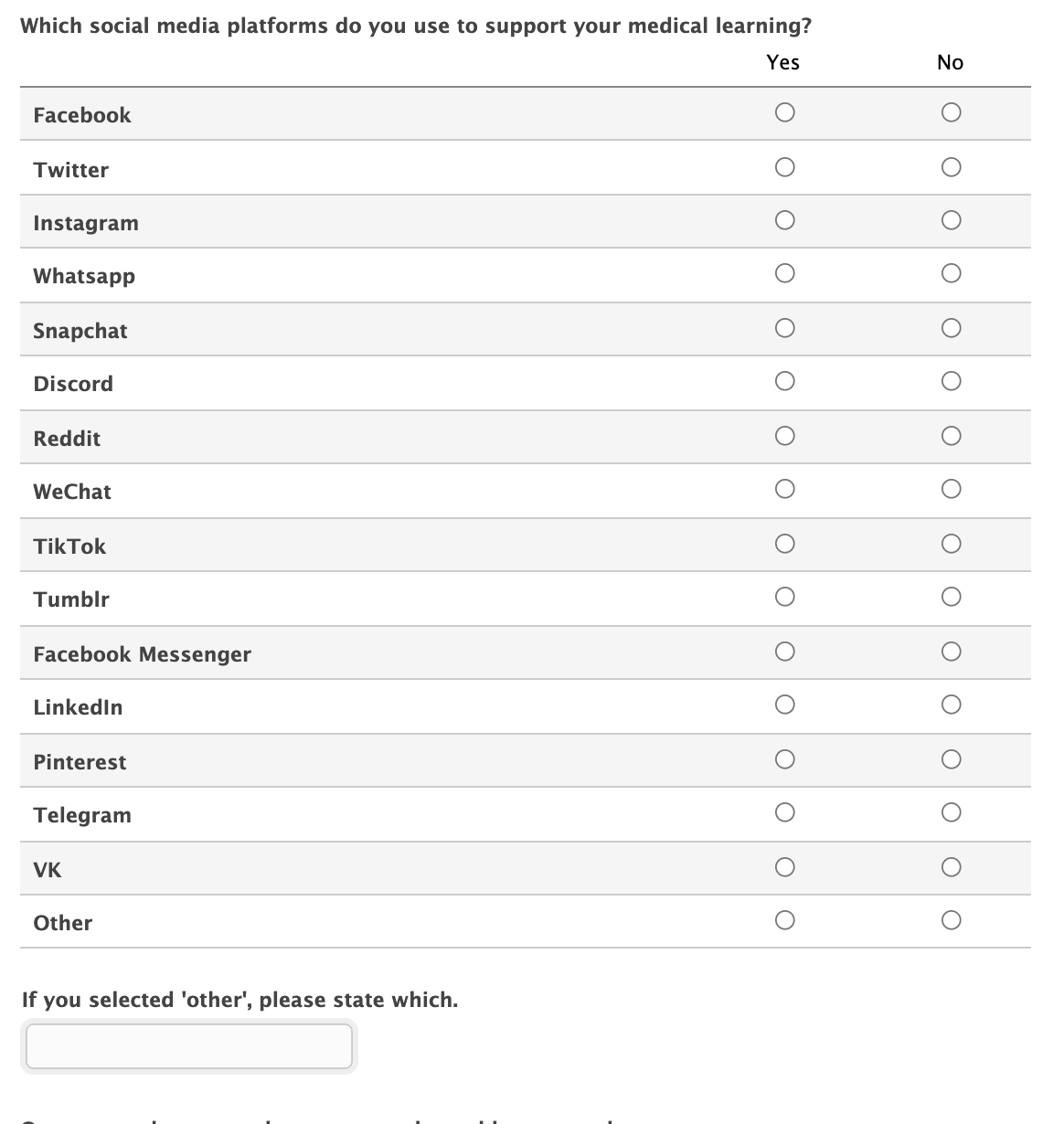


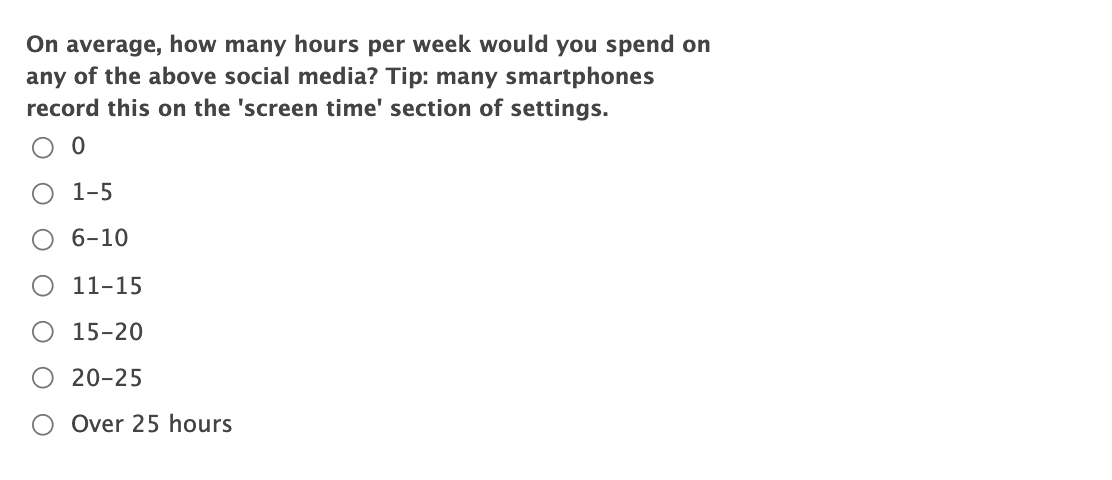


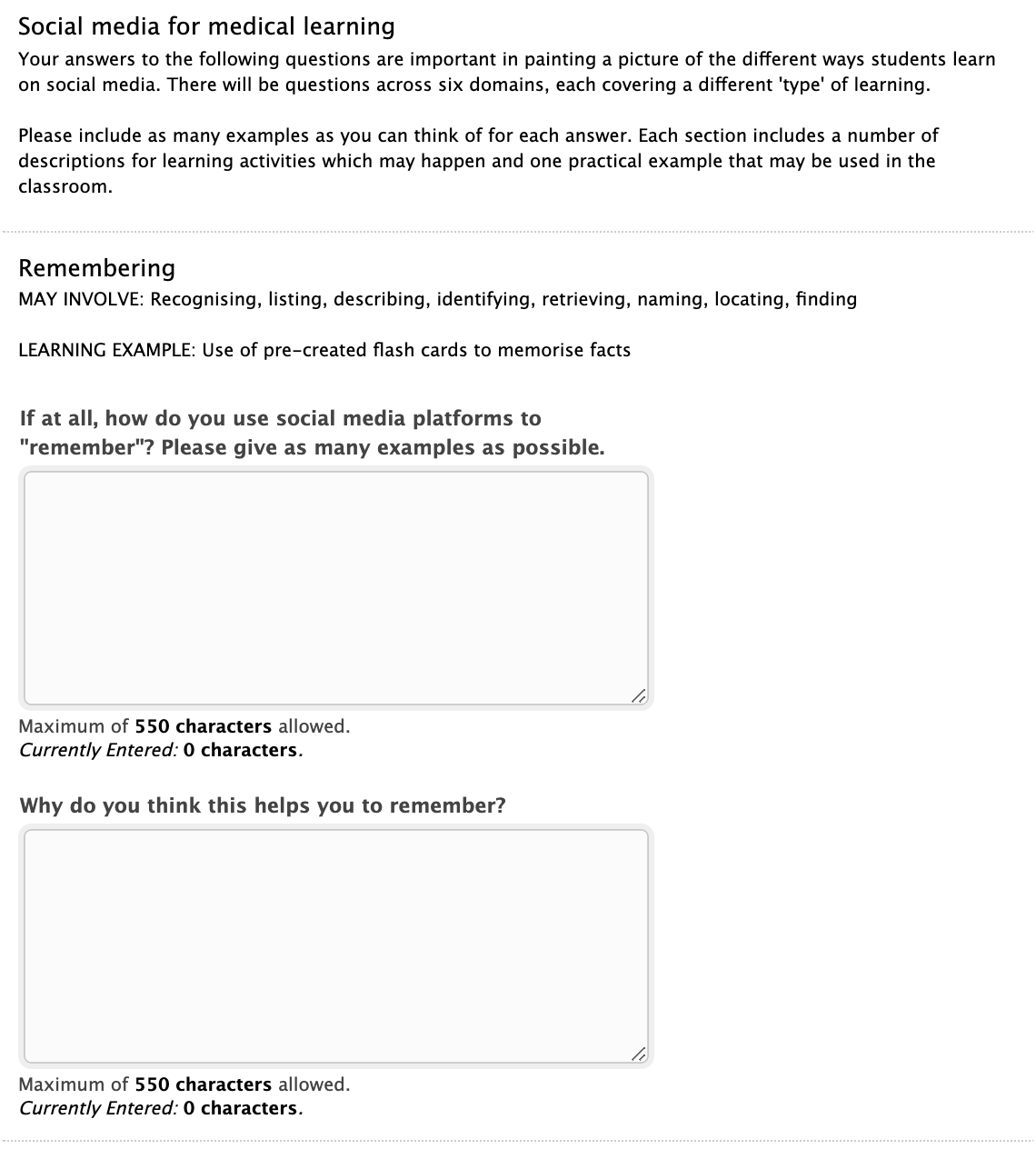


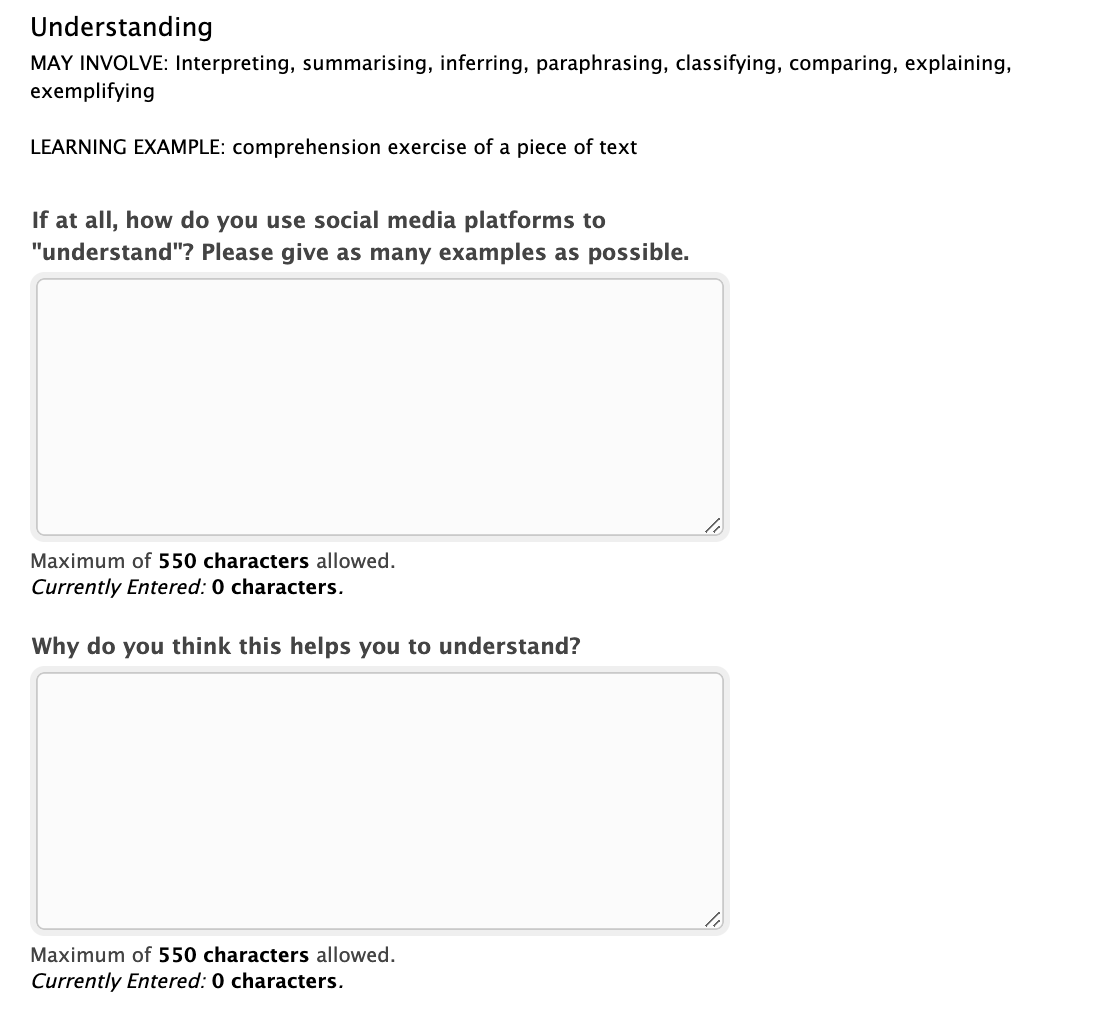


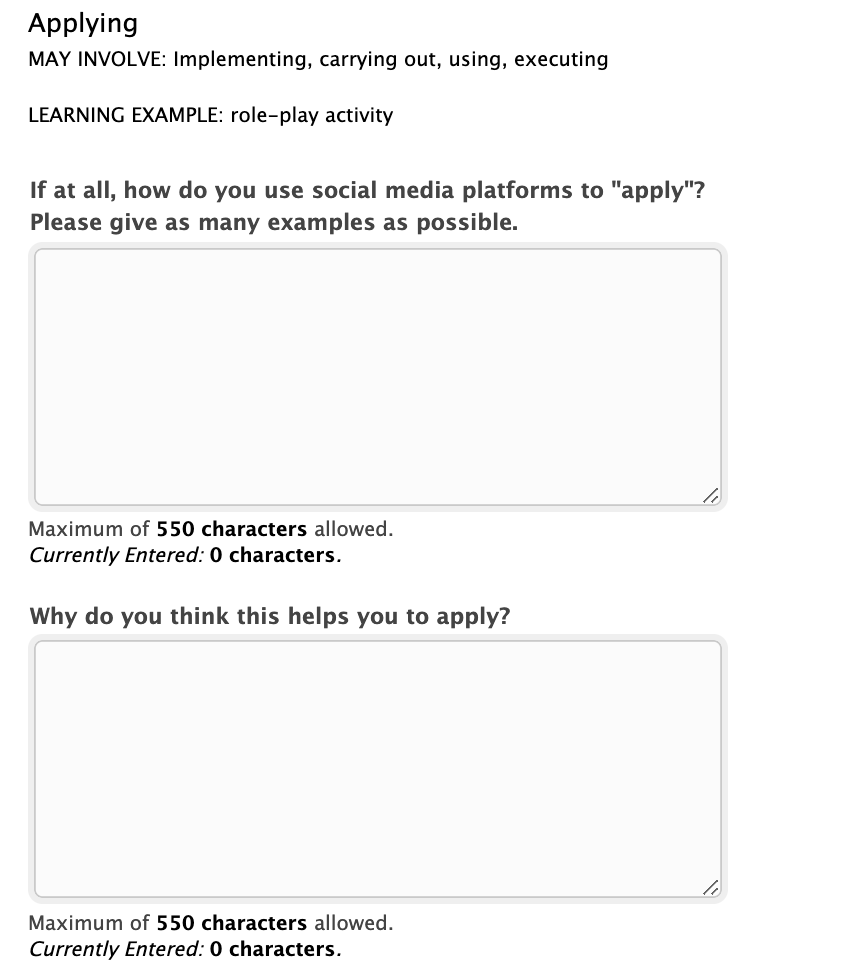

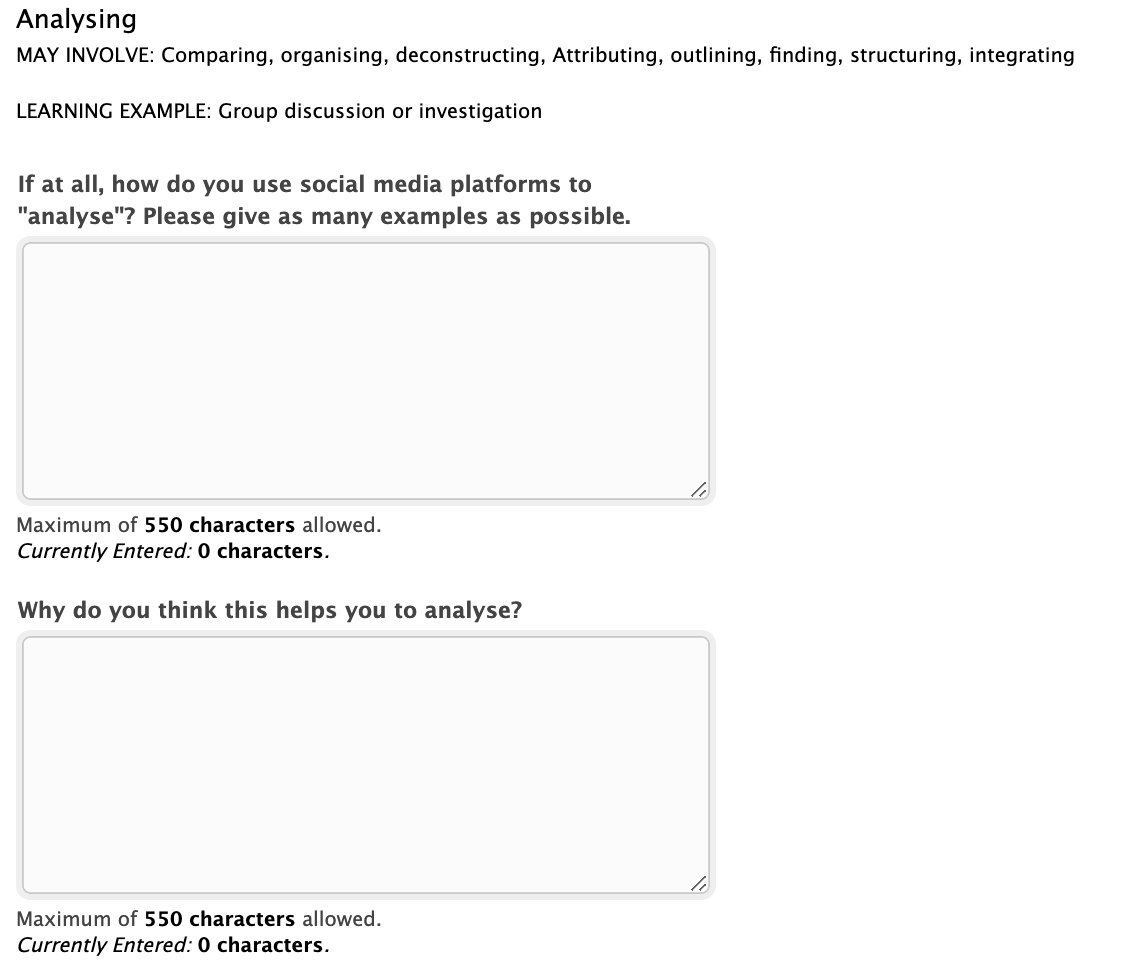


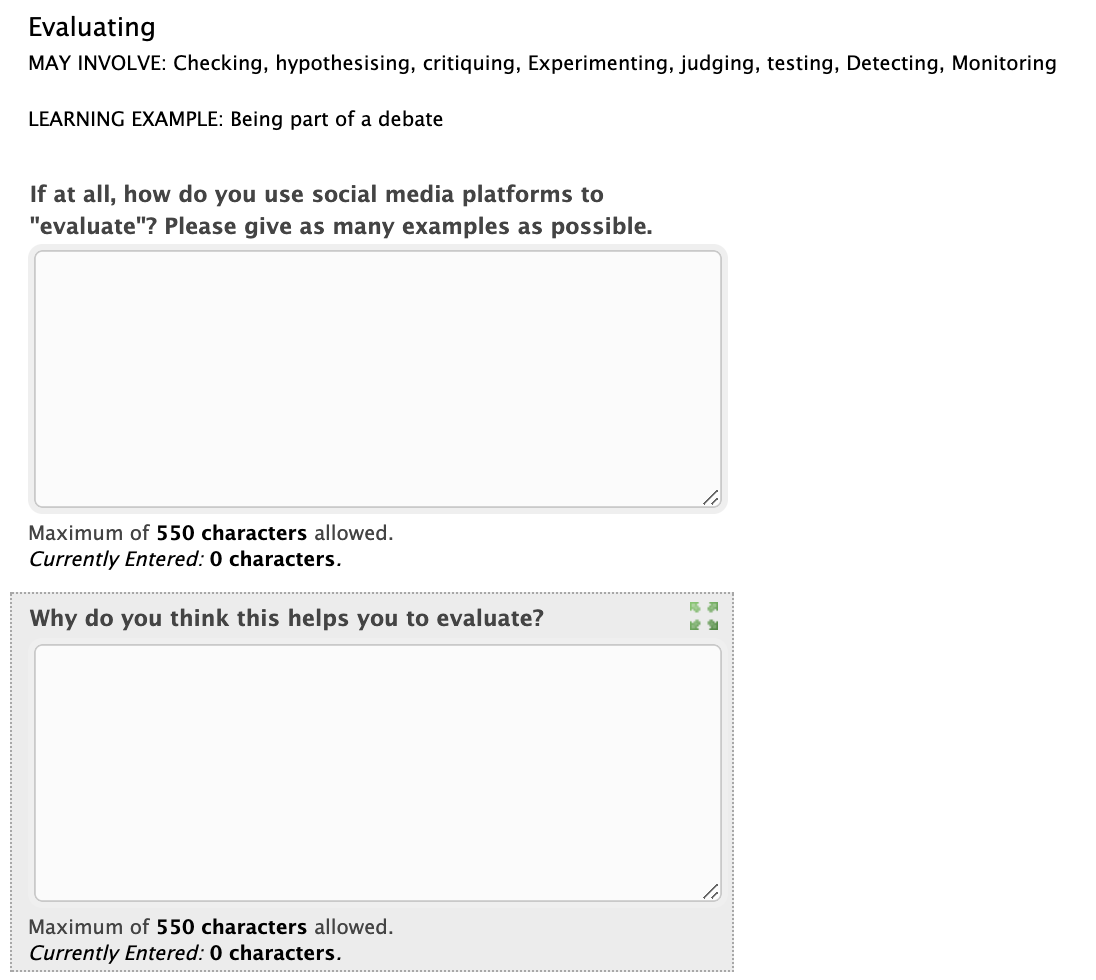

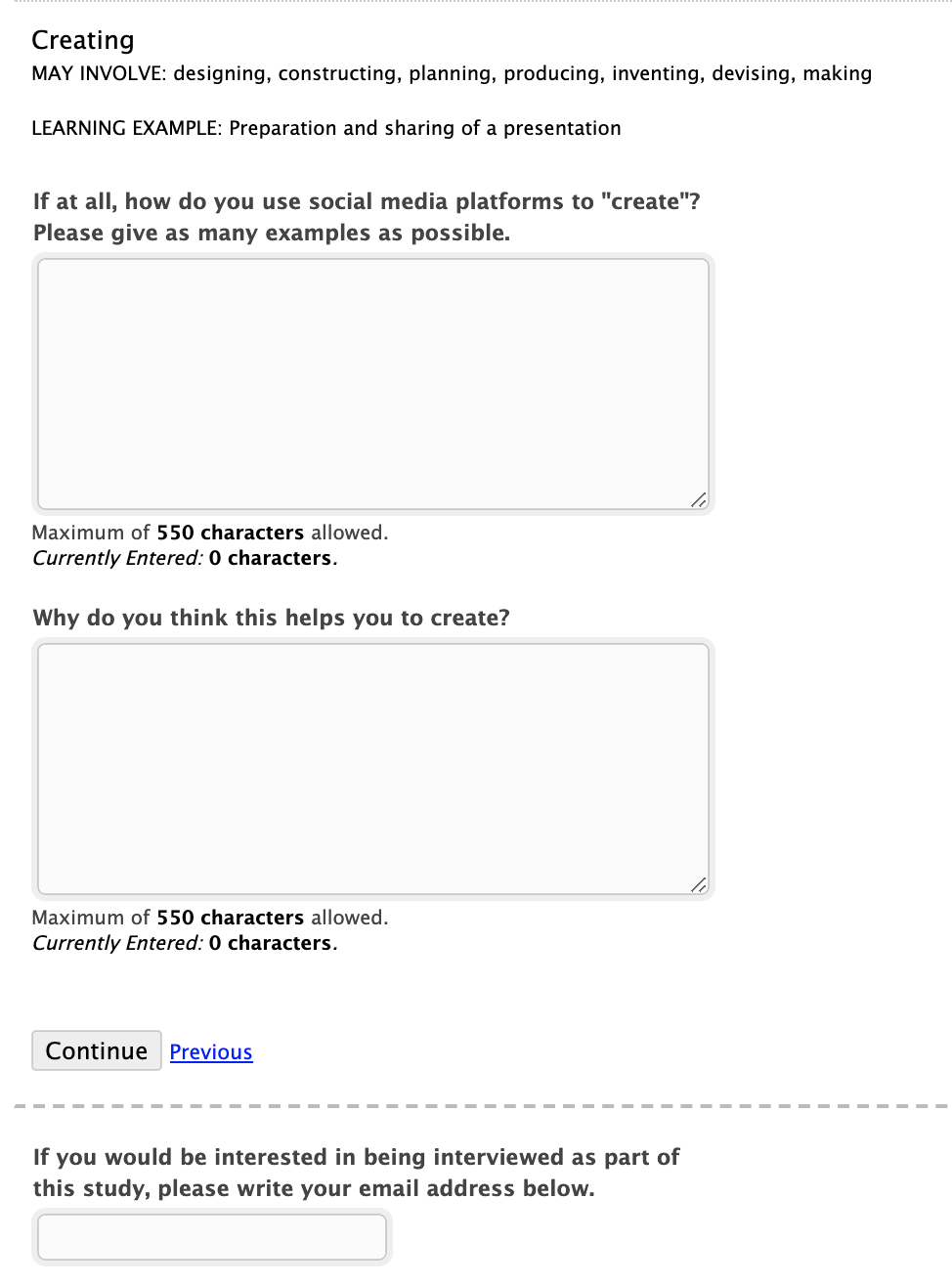

Supplement: Supplementary file 1 — Study Questionnaire. [file TCT-22-e13825-s001.docx]
