# Supplementary material for: Social media quality in undergraduate medical education: A reconceptualisation and taxonomy
Source: Clin Teach. 2024 Nov 6;22(1):e13825. doi: 10.1111/tct.13825 (PMC11663730; doi:10.1111/tct.13825)
Supplement: Supplementary file 2 — Interview Schedule Guide. [file TCT-22-e13825-s002.docx]

**Supplementary File 2: Interview Schedule Guide**

| Question |
| --- |
| 1. **Introduction (in brief)**  *My name is [name], I’m [role]. This research aims to understand quality relating to social media learning. The interview will go through your experiences using social media learning and ask for your perspectives on what high or low quality learning might look like on social media. It should last around 1 hour.*  *Please take this opportunity again to look over the information sheet regarding the interview. You can opt out and stop the interview at any time. The interview and research will not affect your studies in any way. If you have any concerns at all please do let me know now or following the interview.*  *Do you consent to proceed with the interview?* ***Explain purpose of recruitment, purpose of study and my SoMe roles to promote power dynamic transparency.*** |
| **To start, what is learning on social media like for you?**  *Which social media accounts do you use?  Can you tell me about how you generally use these?*  *You mentioned ‘X’ platform. Tell me more about that. Why do you use it? What is the difference between platform X and platform Y you mentioned? Tell me about a typical day on social media.* ***If engagement mentioned*** *– What does ‘engagement’ on social media mean to you?* ***If communities mentioned*** *– Who do you learn with on social media?*  ***If aspects of Bloom’s Taxonomy mentioned, consider asking about specific activities:*** *remembering, understanding, applying (providing context, realism), analysing (organising, categorising), evaluating (reflecting, critiquing), creating (making something new)* |
| **Can you tell me about a time you learned something on social media?**  *Why did it work?  Have you used this method elsewhere on SoMe or affected how you learn more generally? What drove you to choose this method? Tell me about X aspect. How did that help your learning? Talk me through how you learned in this way. You said you felt/experienced X. What was it about social media that made you feel/experience this?* ***If ‘quality’ touched upon –*** *What does ‘quality’ mean to you on social media?  Can some platforms or activities be ‘better’ than others on social media?* ***If struggling to come up with example - *PAUSE, GIVE TIME* -*** *What was the last vaguely educational thing you did on social media?*  ***If aspects of Bloom’s Taxonomy mentioned, consider asking about specific activities:*** *remembering, understanding, applying (providing context, realism), analysing (organising, categorising), evaluating (reflecting, critiquing), creating (making something new)* |
| **Imagine the perfect social media learning resource for you. What would it look like?**  *Are there any learning activities on social media which may help you learn ‘better’ than others?*  *Why does X work/not work for you?* *What does a low quality resource look like to you?  How can you tell that a resource or source is high or low quality?* ***If struggling to describe - *PAUSE, GIVE TIME* -*** *this could be any platform that exists, or any platform you’d like to exist. It could also be an activity, rather than a platform. I’m just interested to know what you like. Are there any values an activity or platform might embody which might help with your learning? Do you think that some learning is better than others in terms of quality?  What do you enjoy about learning on SoMe?* ***If aspects of Bloom’s Taxonomy mentioned, consider asking about specific activities:*** *remembering, understanding, applying (providing context, realism), analysing (organising, categorising), evaluating (reflecting, critiquing), creating (making something new)* |
| ***Thinking back to times that you have learned from SoMe, which factors do you think made these high quality?*** *How might communities or peers impact the quality of your learning?  What about whether a resource is public or private? Are there any factors that would make you wish to learn publicly or privately? Do you think that some learning is better than others in terms of quality?*  *How might audiences on SoMe affect your learning? Do you think that you can have a learning culture on social media?* ***If yes:*** *What might that look like or feel like? Do you feel your medical school provides this kind of learning? Do you interact with experts on social media? How does your medical school act on social media? Have you ever encountered or undertaken any learning activities run by university faculty on social media? Has COVID-19 affected your learning on social media?* ***If Identity mentioned*** *– What kind of person do you think you are on social media? Is this different to who you are in ‘real life’?  Do you act differently in real life compared to social media? Do you act differently in private social media compared to public social media?*  **Can you tell me about any times when you have tried to learn on social media, but it hasn’t gone very well?**  *Why do you think this happened?  How did this make you feel? How did this affect your social media learning more generally?  Did you feel supported? How do you respond to challenge on SoMe? Are you regularly challenged?* ***If professionalism mentioned:*** *How would you define ‘professionalism’ on social media? Does this affect your learning? Have you ever witnessed any unprofessional behaviour on social media? What might make you engage or disengage with social media? Do you think you can learn as effectively by ‘lurking’ on social media as you might do through active participation? Have your social media learning behaviours changed at all over time?* ***If influencers mentioned:*** *What are influencers? How might they affect your learning?* ***If trustworthiness mentioned:*** *Do you ever double check to see if information is correct off social media? What makes you trust someone on social media? Do follower counts impact upon reliability? Is an expert with fewer followers more reliable than a student with many followers?* |
| **Debrief**  *Is there anything important we haven't covered or anything else you would like to discuss with me?*  *Would you like some time following ending the recording to discuss any of the issues raised?* ***Discuss member checking*** |
